# Electroacupuncture Prevents Against AD‐Like Phenotypes in APP/PS1 Mice: Investigation of the Mechanisms From Cerebral Microangiopathy

**DOI:** 10.1002/cns.70696

**Published:** 2025-12-09

**Authors:** Chen Yang, Baobao Li, Shaojie Yang, Xuncui Wang, Guoqi Zhu, Jingji wang

**Affiliations:** ^1^ Center for Xin'an Medicine and Modernization of Traditional Chinese Medicine of IHM, and Key Laboratory of Molecular Biology (Brain Diseases) Anhui University of Chinese Medicine Hefei China; ^2^ Key Laboratory of Xin'an Medicine The Ministry of Education Hefei China; ^3^ The Second Affiliated Hospital of Anhui University of Chinese Medicine Hefei China

**Keywords:** AD‐like phenotype, APP/PS1, cerebral blood flow, electroacupuncture, microangiopathy

## Abstract

**Background:**

Electroacupuncture (EA) has been widely used in Alzheimer's disease (AD) treatment. However, its underlying mechanisms remain poorly elucidated.

**Purpose:**

This study aimed to investigate the effects of EA on AD‐like phenotypes and explore the mechanisms.

**Methods:**

We first evaluated AD‐like behaviors and cerebral blood flow (CBF) changes in APP_swe_/PS1dE9 (APP/PS1) mice at different ages. Subsequently, the therapeutic effects of EA at acupoints *Baihui* (GV20), *Guanyuan* (CV4), and *Zusanli* (ST36), as well as sunitinib, a PDGFRβ‐specific inhibitor, on AD‐like phenotypes in APP/PS1 mice were investigated. CBF was monitored by laser speckle imaging, and hippocampal synaptic ultrastructure and microvascular morphology were examined by transmission electron microscopy (TEM). Western blot was performed to measure related protein expression. Finally, functional ultrasound (fUS) imaging was used to assess changes in brain‐wide functional connectivity.

**Results:**

Compared with age‐matched wild‐type (WT) mice, 6‐ and 9‐month‐old APP/PS1 mice exhibited significant cognitive decline, while all age groups (3‐, 6‐, and 9‐month‐old) of APP/PS1 mice showed significantly reduced CBF. APP/PS1 mice showed elevated expression of microvascular markers in both the hippocampus and cortex. EA significantly ameliorated AD‐like behaviors and prevented CBF reduction as well as microvascular deformation in 6‐month‐old APP/PS1 mice compared with non‐treatment group. TEM and western blot analysis revealed damaged synaptic structure and reduced synaptic proteins in APP/PS1 mice, all of which were markedly alleviated by EA treatment. In addition, EA treatment downregulated the aberrantly elevated expression of PDGFRβ and CD31, enhanced the levels of tight junction proteins (Occludin, Claudin‐5, and ZO‐1) and glucose transporter 1 (GLUT1), and suppressed the expression of inflammatory proteins. Of note, intervention with sunitinib also improved AD‐like behaviors in APP/PS1 mice. Remarkably, fUS imaging results showed that EA enhanced the functional connection between hippocampal regions of APP/PS1 mice.

**Conclusion:**

Our data demonstrates that EA ameliorates AD‐like phenotypes, potentially through preventing microangiopathy.

## Graphical abstract

Electroacupuncture at combined acupoints GV20, CV4, and ST36 ameliorates AD‐like phenotypes, potentially through preventing microangiopathy and enhancing CBF. These findings provide novel insights for early intervention strategies to halt cognitive decline in AD.

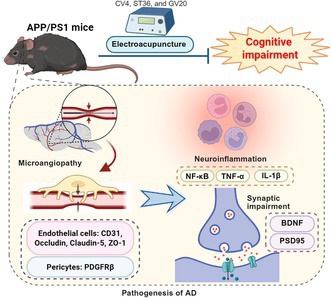

**Abbreviations**
AD: Alzheimer's disease
ADRD: AD‐related dementia
AI: agranular insular area
APP/PS1: APP_swe_/PS1dE9
AUD: Auditory areas
BBB: blood–brain barrier
BPS: Brain Positioning System
CBF: cerebral blood flow
CBV: cerebral blood volume
CSVD: cerebral small vessel disease
CV4: *Guanyuan*
DI: Discrimination Index
DMSO: Dimethyl Sulfoxide
EA: electroacupuncture
ECT: Ectorhinal area
ET: endothelin
FST: Forced swimming test
fUS: function ultrasound imaging
GLUT1: glucose transporter 1
GV20: *Baihui*
GV24: *Shenting*
GWAS: genome‐wide association study
H&E: hematoxylin and eosin
IL‐1β: Interleukin‐1 beta
MWM: Morris water maze
NF‐κB: Nuclear factor kappa‐light‐chain‐enhancer of activated B cells
NOR: novel object recognition
NVU: neurovascular unit
OLF: Olfactory areas
PDGFRβ: platelet‐derived growth factor receptor beta
RHP: Retrohippocampal region
ROS: reactive oxygen species
SSp: Primary somatosensory area
ST36: *Zusanli*
TCM: traditional Chinese medicine
TEM: transmission electron microscope
TH_Poly: Thalamus polymodal association cortex related
TH_SM: Thalamus sensory motor cortex related
TNF‐α: Tumor necrosis factor alpha
TST: Tail suspension test
VD: Vascular Dementia
VIS: visual areas
VP: Pallidum ventral region
WT: Wild‐type
ZO‐1: Zonula occludens‐1

## 1 Introduction

Alzheimer's disease (AD) has emerged as a critical global public health challenge, accounting for 60%–80% of dementia cases. Epidemiological data indicate that approximately 18.1% of individuals aged 65 years and older are affected by AD, with this prevalence rising to 33.2% in the population aged 85 years and above [1]. The clinical manifestations of AD primarily involve cognitive dysfunction accompanied by executive impairment and neuropsychiatric symptoms. Notably, mood disorders may emerge even prior to the onset of cognitive impairment and memory loss [2, 3], representing one of the early AD phenotypes. For decades, drug development for AD prevention and treatment has predominantly focused on late‐stage pathological hallmarks such as β‐amyloid (Aβ) plaques and neurofibrillary tangles, yet most clinical trials have ultimately failed [4].

Emerging evidence in recent years has demonstrated that cerebral capillary changes occur in the early stages of AD [5]. Reduced cerebral blood flow (CBF), recognized as both an early pathological marker and persistent symptom of AD, precedes the clinical symptoms such as Aβ plaque deposition and cognitive impairment and is a critical factor in cognitive decline [6, 7]. Mechanistic study has revealed that Aβ induces cerebral capillary constriction through a cascade involving reactive oxygen species (ROS)‐mediated endothelial endothelin‐1 (ET‐1) release, which activates ET receptors in pericytes. This microcirculatory dysfunction precipitates a cerebral metabolic crisis, directly contributing to cognitive impairment [8]. Therefore, improving the abnormal reduction of CBF in the early stages of AD has emerged as a novel therapeutic target for halting disease progression.

Acupuncture, a widely recognized alternative therapy, has been applied for thousands of years. Among its various techniques, electroacupuncture (EA) is particularly prominent in modern clinical practice due to its standardized operation and quantifiable parameters [9]. Studies have demonstrated that EA at acupoints *Shenting* (GV24) and *Baihui* (GV20) improves memory impairment in early AD mice [10, 11]. Moreover, combined EA stimulation of GV20, GV24, and *Zusanli* (ST36) not only ameliorates anxiety‐like behaviors and memory deficits, but also significantly reduces pro‐inflammatory cytokine levels [12]. Based on the traditional Chinese medicine (TCM) meridian theory, the three acupuncture points GV20, *Guanyuan* (CV4), and ST36 could form a collaborative treatment system of “tonifying the lower (CV4)‐regulating the middle (ST36)‐awakening the upper (GV20)”. It can not only regulate the mind, but also strengthen the foundation and cultivate the essence, thereby achieving the treatment of AD‐like phenotype. However, there is still a long way to go before the mechanism of EA's effect on AD can be fully elucidated.

In this study, we utilized APP_swe_/PS1dE9 (APP/PS1) mice of different ages and first demonstrated that cerebral microvascular dysfunction occurs prior to cognitive impairment. Then we further investigated the therapeutic effects of EA at acupoints GV20, CV4, and ST36 on AD‐like phenotypes and explored the underlying mechanisms. Our findings provide potential experimental evidence for clinically applying EA to intervene in early AD symptoms.

## 2 Materials and Methods

### 2.1 Animals and Treatments

APP/PS1 mice were obtained from Hangzhou Ziyuan Experimental Animal Technology Co. Ltd. [Production License No: SCXK (Zhe) 2019–0004]. To exclude the effect of gender differences on AD‐related cognitive impairment [13], only male mice were used in this study. The mice were housed under controlled conditions: 55% ± 10% relative humidity, 22°C ± 2°C room temperature, and a 12 h/12 h light/dark cycle with *ad libitum* access to food and water. All experimental procedures were approved by the Animal Ethics Committee of Anhui University of Chinese Medicine (Approval No.: AHUCM‐mouse‐2023146).

For EA stimulation, 6‐month‐old APP/PS1 mice were delivered EA treatment at acupoints GV20, CV4, and ST36. GV20 was located at the middle of the parietal bone, CV4 approximately 10 mm inferior to the umbilicus, and ST36 on the posterior‐lateral aspect of the hindlimb knee joint, approximately 2 mm distal to the fibular head [14]. After anesthesia with 5% isoflurane (RWD Life Science, China), the mice were fixed in a lateral position, exposed the acupoints, and locally disinfected. Then, the disposable acupuncture needles (diameter 0.20 mm, length 13 mm) were inserted into the corresponding acupoints: GV20 at 45° forward‐diagonal to 2‐mm depth, CV4 perpendicular to 2‐mm depth, and ST36 perpendicular to 4‐mm depth. Needles were connected to an electronic acupuncture apparatus (Huatuo, China) with GV20 (cathode) paired to ipsilateral ST36 (anode), and CV4 (cathode) to contralateral ST36 (anode). Electrical stimulation was delivered at 3 Hz and 1 mA intensity for 30 min per session, administered once daily for 14 consecutive days.

**FIGURE 1:**
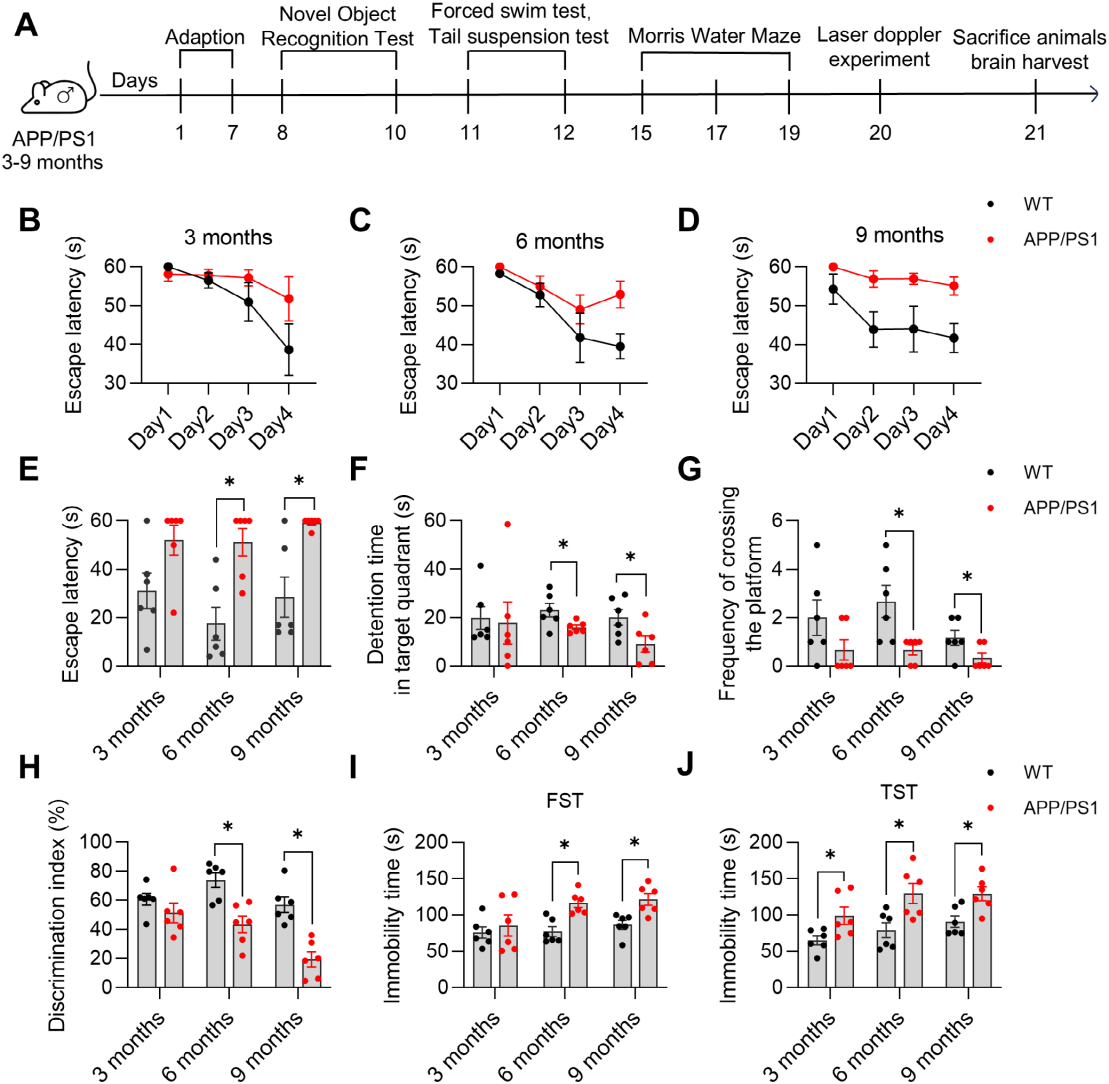
Age‐dependent changes in cognitive function in APP/PS1 mice. (A) Experimental flowchart for *Experiment 1*. (B‐D) Escape latencies during the spatial navigation trial. (E) The escape latency during the spatial probe trial. (F) The time spent in the target quadrant during the spatial probe trial. (G) Frequency of crossing the platform in the target quadrant during the spatial probe trial. (H) Discrimination index at different ages in the new object recognition test. (I) Immobility time in the forced swim test. (J) Immobility time in the tail suspension test. Data are expressed as mean ± SEM (*n* = 6 per group). *p < 0.05 between groups. (Independent student *t*‐test).

*Experiment 1*: APP/PS1 mice at 3‐, 6‐, and 9‐month‐old and age‐matched wild‐type (WT) mice were divided into 6 groups according to the age of the mice (*n* = 6 per group): 3 months WT group, 3 months APP/PS1 group, 6 months WT group, 6 months APP/PS1 group, 9 months WT group, and 9 months APP/PS1 group. Following 1 week of acclimatization, behavioral tests including novel object recognition, Morris water maze, tail suspension test, and forced swim test were conducted simultaneously with CBF assessment. Subsequently, mice were deeply anesthetized with 5% isoflurane inhalation and euthanized via decapitation for brain collection (Figure 1A).

**FIGURE 2:**
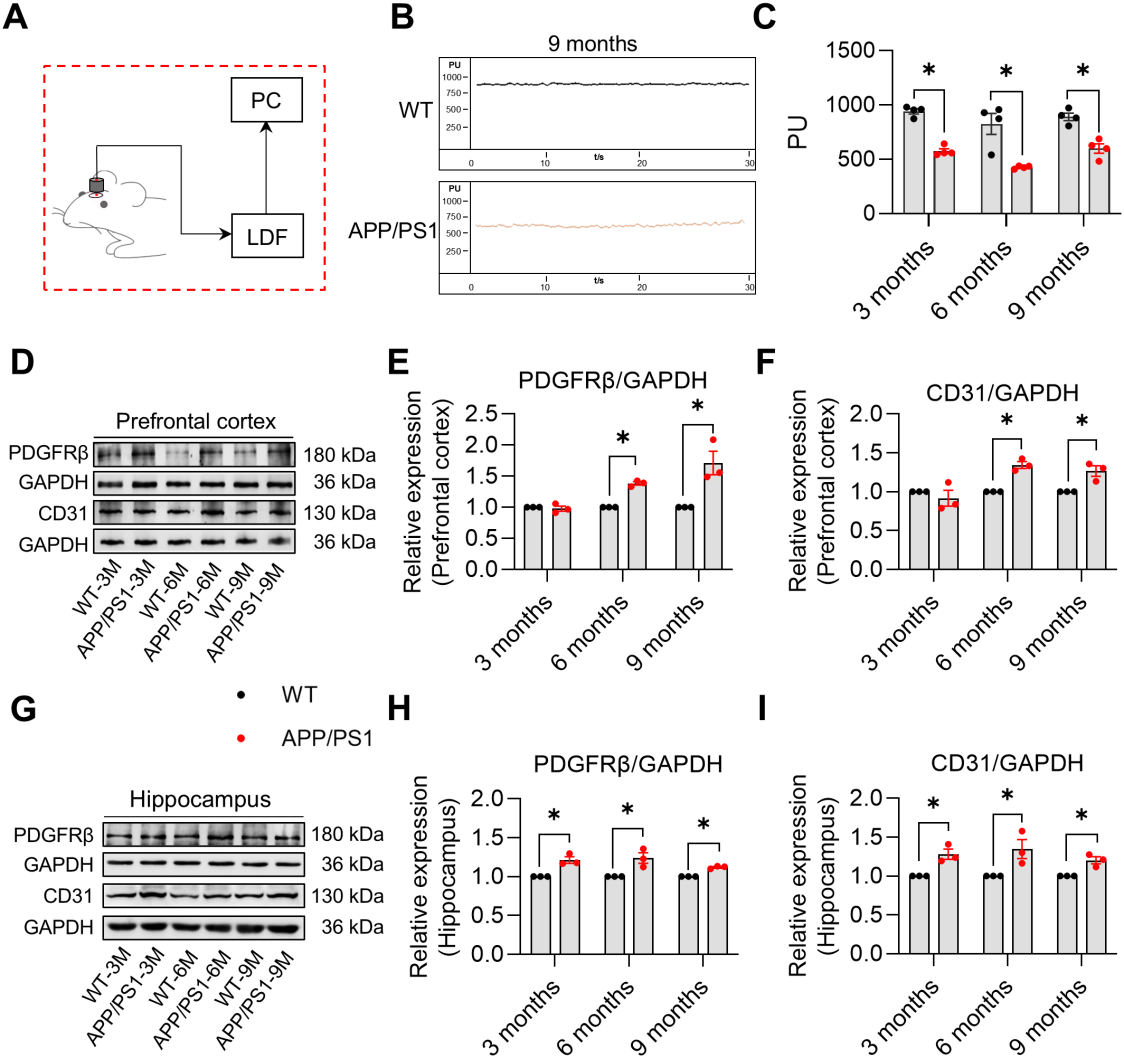
Changes in CBF and expression of microvascular markers in APP/PS1 mice aged 3, 6, and 9 months. (A) Schematic diagram of laser doppler blood flowmetry for cerebral blood flow measurement. (B) Representative laser doppler flowmetry images showing cerebral blood flow changes in the cortex of 9‐month‐old mice. (C) Quantitative data of cerebral blood flow changes in the cortex of 3‐, 6‐, and 9‐month‐old mice. (D) Representative immunoblots of PDGFRβ and CD31 expression in the prefrontal cortex. (E) Quantitative analysis of PDGFRβ expression in the prefrontal cortex of 3‐, 6‐, and 9‐month‐old mice. (F) Quantitative analysis of CD31 expression in the prefrontal cortex of 3‐, 6‐, and 9‐month‐old mice. (G) Representative immunoblots of PDGFRβ and CD31 expression in the hippocampus. (H) Quantitative analysis of PDGFRβ expression in the hippocampus of 3‐, 6‐, and 9‐month‐old mice. (I) Quantitative analysis of CD31 expression in the hippocampus of 3‐, 6‐, and 9‐month‐old mice. Data are expressed as mean ± SEM (*n* = 4 or 3 per group). *p < 0.05 between groups. (Independent student's *t*‐test).

*Experiment 2*: Based on the results from *Experiment 1*, 6‐month‐old APP/PS1 mice exhibited cognitive impairment and cerebral microangiopathy, and thus this age group of mice was selected for subsequent investigations. 6‐month‐old APP/PS1 mice (hereafter referred to as APP/PS1) and age‐matched WT mice were randomly divided into 4 groups (*n* = 10 per group): WT group, APP/PS1 group, APP/PS1 + EA group, and APP/PS1 + Donepezil (Donep) group. APP/PS1 + EA mice received daily EA for 14 consecutive days, followed by behavioral tests. APP/PS1 + Donep mice were administered donepezil (5 mg/kg) once daily via oral gavage for 14 days. The donepezil dosage was determined based on a previous study [15]. WT, APP/PS1, and APP/PS1 + Donep mice underwent the same inhalation of anesthesia as the APP/PS1 + EA group. Additionally, WT and APP/PS1 mice received daily distilled water gavage for 14 days (Figure 2A).

*Experiment 3*: According to our previous publication [15], we administered sunitinib (S1042, Selleck Chemicals, USA), a PDGFRβ inhibitor targeting cerebral microvascular pericytes. 6‐month‐old APP/PS1 mice were randomly divided into 2 groups (*n* = 4 per group): APP/PS1 group and APP/PS1 + Sunitinib group. The APP/PS1 + Sunitinib mice were given sunitinib (40 mg/kg) intraperitoneal injection once a day for 14 days. The APP/PS1 group was given equal amounts of normal saline.

### 2.2 Behavioral Tests

All behavioral tests were conducted following the completion of the treatment period. In *Experiment 1* (*n* = 6), a *t*‐test was employed to compare APP/PS1 mice of different ages with their age‐matched WT mice. The data from *Experiment 2* (*n* = 10) were analyzed using one‐way analysis of variance (ANOVA), while the *t*‐test was again applied in *Experiment 3* (*n* = 4). To ensure objectivity and eliminate bias, all behavioral assessments were strictly performed under double‐blind conditions.

#### 2.2.1 Novel Object Recognition (NOR) Test

The NOR test was conducted to evaluate cognitive function in mice using a three‐period protocol: habituation, training, and testing. Animal movements were tracked using SuperMaze Animal Behavior Analysis Software (Shanghai Xinruan Technology, China) via an overhead camera. During the 5 min habituation, mice were gently placed in the center of an open‐field arena (400 × 400 × 400 mm) for free exploration. During the training period (24 h after the end of habituation), two identical objects without special odor were placed in two opposite positions in the open‐field arena, and the mice were allowed to freely explore the open‐field arena for 10 min. During the testing period (24 h after the end of training), one of the training objects was replaced with another new object without special odor. The mice were put into the open‐field arena to explore freely for 5 min, and activity traces were recorded.

#### 2.2.2 Morris Water Maze (MWM) Test

The MWM test was conducted to evaluate spatial learning and memory in mice. The apparatus consisted of a circular pool (diameter 100 cm, depth 30 cm) maintained at 22°C ± 2°C. The pool was conceptually divided into four quadrants, with a hidden platform (diameter 10 cm) positioned in the center of the fourth quadrant. The spatial navigation trial spanned 4 days, during which the concealed platform was consistently positioned in the fourth quadrant, approximately 2 cm beneath the water's surface. The swimming behavior and escape latency of mice were recorded and tracked within 60 s. If the mouse failed to locate the platform within 60 s, it was gently guided to the platform and permitted to remain there for 10 s. During the probe trial, the platform was removed and mice were gently placed into the water, oriented towards the wall of the second quadrant. The escape latency, frequency of crossing the platform, and the time spent in the target quadrant were recorded within 60 s. Subsequent data analysis was performed using the EthoVision XT software (Noldus, Netherlands).

#### 2.2.3 Tail Suspension Test (TST)

The TST was used to evaluate the behavioral despair in mice as previously described [16]. Mice acclimating to the testing environment for 24 h were gently removed from their cages and securely fastened 2 cm from the tail tip without applying excessive pressure or inducing stress. Each session was video recorded for 6 min, with immobility duration during the final 4 min being quantified.

#### 2.2.4 Forced Swimming Test (FST)

The FST was used to evaluate the behavioral despair in mice as previously described [17]. After 24 h environmental acclimation, mice were gently grasped at the mid‐tail region and slowly lowered into 24°C water. Each 5 min session was video‐recorded, with immobility duration during the final 4 min quantified.

### 2.3 Laser Doppler Blood Flowmetry

After anesthesia with 5% isoflurane, mice were secured in a stereotaxic frame. The scalp was routinely disinfected, and the meninges were carefully separated to fully expose the skull between the coronal and lambdoid sutures. Cerebral cortical blood flow was continuously monitored for 30 min using laser Doppler blood flowmetry (PROBE 407, Perimed, Sweden) positioned 1.8 mm posterior to bregma and 0.35 mm lateral to the midline, with data acquisition through PSW software (Perimed, Sweden). Throughout the recording session, all potential light interference from both natural and ambient sources was strictly avoided.

### 2.4 Laser Speckle Imaging

Mice were anesthetized with 5% isoflurane and immobilized in a stereotaxic apparatus. Following standard scalp disinfection, a midline incision was made and the meninges were gently retracted to expose the cranial surface between the coronal and lambdoid sutures. Real‐time CBF monitoring was performed using a laser speckle contrast imaging system (RWD, China) configured with a 20 ms exposure time, 5 s temporal filtering, time‐dependent analysis algorithm, 0.2 Hz acquisition rate, and 10% background threshold intensity. The imaging probe was maintained 20–25 cm above the skull, providing a 5 × 5 cm field of view for real‐time perfusion monitoring.

### 2.5 Functional Ultrasound (fUS) and Ultrafast Ultrasound Imaging (ULM)

Following established protocols [18], mice were initially anesthetized with isoflurane and secured in a stereotaxic frame. After skull exposure, the cranial surface was cleaned and mechanically thinned, followed by rigid fixation of the head‐holding apparatus using dental acrylic cement. Postoperatively, animals were rewarmed for recovery while a 3D‐printed protective cover was affixed to the head frame to maintain surgical site sterility.

Before experiments, the protective cover was removed, and mice were sedated via subcutaneous medetomidine injection. They were then head‐fixed on a custom treadmill, with ultrasound coupling gel applied to ensure optimal acoustic contact between the skull and transducer. The imaging plane was determined using standard hippocampal coordinates and real‐time cerebral blood volume (CBV) imaging for probe alignment. Following angiographic scanning, data were registered to the Allen Mouse Brain Atlas using IcoStudio software, with precise targeting via the Brain Positioning System (BPS). 3D fUS imaging was performed using a 15 MHz transducer (IcoPrime‐15 MHz, 256 elements, center frequency 15 MHz, spatial resolution 100 × 100 μm^2^, Iconeus, Paris, France) connected to an ultrafast ultrasound scanner (Iconeus One, 256 channels, Iconeus, Paris, France). The probe was mounted on a linear motorized stage (SLC‐1740, SmarAct GmbH) with a 26 mm travel range. Data were acquired for 40 min using the integrated IcoStudio software (Iconeus, Paris, France). CBV signals were extracted from 102 brain regions of interest (ROIs) to quantify functional connectivity patterns. Resting‐state functional connectivity of transcranial volumes was assessed under medetomidine sedation.

Microbubbles (MBs, WeView, China) were intravenously injected (0.1 mL/mouse) via the tail vein in anesthetized thinned‐skull mice. ULM data were acquired for 10 min using the Iconeus One ultrasound system and processed using SVD‐based spatiotemporal filtering to isolate MB signals from tissue background. MBs were identified as local intensity maxima in filtered images. Maximum intensity projections of MB trajectories in individual coronal/sagittal planes were generated using “Compute SuperLoc” (Iconeus One), with key data extracted via “Display SuperLoc”. Functional connectivity data were analyzed using MATLAB (R2023b). Statistical comparisons across the three groups were performed by applying Fisher's Z‐transformation to correlation coefficients, followed by the nonparametric Kruskal‐Wallis H‐test. Where significant overall differences were detected, post hoc pairwise comparisons were conducted using Dunn's test with appropriate correction for multiple comparisons.

### 2.6 Western Blotting

Prefrontal cortex and hippocampal tissues were homogenized in 100 μL RIPA lysis buffer supplemented with 1 μL PMSF, incubated on ice for 30 min, and centrifuged at 12,000 rpm (4°C, 5 min). The supernatants were collected, and protein concentrations were determined using a BCA assay kit. After that, protein samples were separated by SDS‐PAGE and transferred to nitrocellulose membranes (400 mA, 30–40 min) at room temperature. After blocking with 5% non‐fat milk for 2 h at room temperature, the membranes were incubated with primary antibodies overnight at 4°C: GAPDH (1:1000, BM3874, Boster, China), platelet‐derived growth factor receptor beta (PDGFRβ) (1:1000, A00096‐1, Boster, China), Zonula occludens‐1 (ZO‐1) (1:1000, PB9234, Boster, China), Claudin 5 (1:1000, 29,767–1‐AP, Proteintech, China), Occludin (1:1000, A01246‐4, Boster, China), NF‐κB (1:1000, CY5034, Abways, China), TNF‐α (1:1000, RM8040, Biodragon, China), IL‐1β (1:1000, CY5087, Abways, China), BDNF (1:1000, E17G19, Selleck, China), PSD95 (1:1000, F17C8, Selleck, China), CD31 (1:1000, 11,265–1‐AP, Proteintech, China), glucose transporters 1 (GLUT1) (1:1000, P22E15, Selleck, China). Following three 10‐min washes with PBST containing 0.05% Tween 20, the membranes were incubated with HRP‐conjugated goat anti‐rabbit IgG or anti‐mouse IgG (1:10,000; Zs‐bio, China) for 2 h at room temperature. Protein bands were visualized using enhanced chemiluminescence reagent (Tanon, China) and analyzed using ImageJ software.

### 2.7 Transmission Electron Microscope (TEM)

Fresh hippocampal tissues were cut into 1 mm^3^ and fixed in 2.5% glutaraldehyde solution. The samples were then washed with 0.1 mol/L phosphate buffer. After that, they were fixed with 1% osmium acid for 1.5 h. Subsequently, the tissues were methodically dehydrated using a graded series of ethanol and acetone, transitioned into propylene oxide, and embedded in epoxy resin. The embedded samples were then polymerized for 48 h. Hippocampal slices (70 nm thickness) were then prepared using an ultramicrotome (Leica, Germany). These sections were stained with 2% uranyl acetate and lead citrate. After washing and drying, hippocampal slices were observed using transmission electron microscopy (Hitachi, Japan). Statistical analyses of synaptic cleft and PSD thickness were performed in WT, APP/PS1, and APP/PS1 + EA mice (*n* = 6 per group) using Image‐Pro Plus 6.0 software.

### 2.8 Statistical Analysis

Data were expressed as mean ± standard error of the mean (SEM) and analyzed using GraphPad Prism 9.5 software. The data with normal distribution and homogeneity of variances were compared by independent student *t*‐test or one‐way ANOVA followed by Tukey–Kramer test, while those with non‐normal distribution were tested by Kruskal‐Wallis nonparametric statistics, followed by Dunn's test. *p* < 0.05 was considered significant.

## 3 Results

### 3.1 Age‐Dependent Changes in Cognitive Function in APP/PS1 Mice

To investigate the cognitive function in APP/PS1 mice at different months of age, we performed a comprehensive behavioral test including the MWM, NOR, TST, and FST (Figure 1A). The results of MWM showed that the escape latencies of all groups were gradually shortened during the spatial navigation trial (Figure 1B–D). In the spatial exploration trial, 6‐ and 9‐month‐old APP/PS1 mice showed significantly prolonged escape latency (6‐month‐old: *p* < 0.05, *t* = 3.818, df = 10; 9‐month‐old: *p* < 0.05, *t* = 3.671, df = 10, Figure 1E, respectively), decreased detention time in the target quadrant (6‐month‐old: *p* < 0.05, *t* = 2.352, df = 10; 9‐month‐old: *p* < 0.05, *t* = 2.372, df = 10, Figure 1F, respectively), and reduced frequency of crossing the platform (6‐month‐old: *p* < 0.05, *t* = 2.860, df = 10; 9‐month‐old: *p* < 0.05, *t* = 2.236, df = 10, Figure 1G, respectively) compared with age‐matched WT mice. The NOR test revealed a significantly decreased discrimination index in both 6‐month‐old (*p* < 0.05, *t* = 3.993, df = 10) and 9‐month‐old APP/PS1 mice (*p* < 0.05, *t* = 5.016, df = 10) compared with age‐matched WT mice (Figure 1H). However, 3‐month‐old APP/PS1 mice showed no statistically significant differences compared to age‐matched WT mice in both the MWM and NOR tests (*p* > 0.05, Figure 1E–H). In the FST, 6‐ and 9‐month‐old APP/PS1 mice exhibited significantly increased immobility time (6‐month‐old: *p* < 0.05, *t* = 4.275, df = 10; 9‐month‐old: *p* < 0.05, *t* = 3.449, df = 10, Figure 1I, respectively) compared with age‐matched WT mice, while 3‐, 6‐, and 9‐month‐old APP/PS1 mice displayed significantly prolonged immobility time (3‐month‐old: *p* < 0.05, *t* = 2.500, df = 10; 6‐month‐old: *p* < 0.05, *t* = 2.915, df = 10; 9‐month‐old: *p* < 0.05, *t* = 3.058, df = 10, Figure 1J, respectively) in the TST.

### 3.2 Changes in CBF and Expression of Microvascular Markers in APP/PS1 Mice Aged 3, 6, and 9 Months

Laser doppler blood flowmetry revealed significantly reduced CBF in 3‐, 6‐, and 9‐month‐old APP/PS1 mice compared to age‐matched WT mice (3‐month‐old: *p* < 0.05, *t* = 11.200, df = 6; 6‐month‐old: *p* < 0.05, *t* = 4.105, df = 6; 9‐month‐old: *p* < 0.05, *t* = 5.287, df = 6, Figure 2A–C, respectively). Western blot analysis revealed significantly elevated expression of the pericyte marker PDGFRβ (3‐month‐old: *p* > 0.05, *t* = 0.5050, df = 4; 6‐month‐old: *p* < 0.05, *t* = 14.08, df = 4; 9‐month‐old: *p* < 0.05, *t* = 3.766, df = 4, Figure 2D,E, respectively) and the endothelial marker CD31 (3‐month‐old: *p* > 0.05, *t* = 0.8088, df = 4; 6‐month‐old: *p* < 0.05, *t* = 7.848, df = 4; 9‐month‐old: *p* < 0.05, *t* = 3.929, df = 4, Figure 2D,F, respectively) in the prefrontal cortex of 3‐, 6‐, and 9‐month‐old APP/PS1 mice compared with age‐matched WT mice. PDGFRβ (3‐month‐old: *p* < 0.05, *t* = 5.115, df = 4; 6‐month‐old: *p* < 0.05, *t* = 3.497, df = 4; 9‐month‐old: *p* < 0.05, *t* = 11.36, df = 4, Figure 2G,H, respectively) and CD31 (3‐month‐old: *p* < 0.05, *t* = 4.325, df = 4; 6‐month‐old: *p* < 0.05, *t* = 2.865, df = 4; 9‐month‐old: *p* < 0.05, *t* = 4.402, df = 4, Figure 2G,I, respectively) showed a similar increasing trend in the hippocampus. Together with the behavioral results, these data suggest that micropathology precedes cognitive decline and may represent an initial hallmark in AD pathogenesis.

### 3.3 EA Ameliorates AD‐Like Phenotypes in APP/PS1 Mice

**FIGURE 3:**
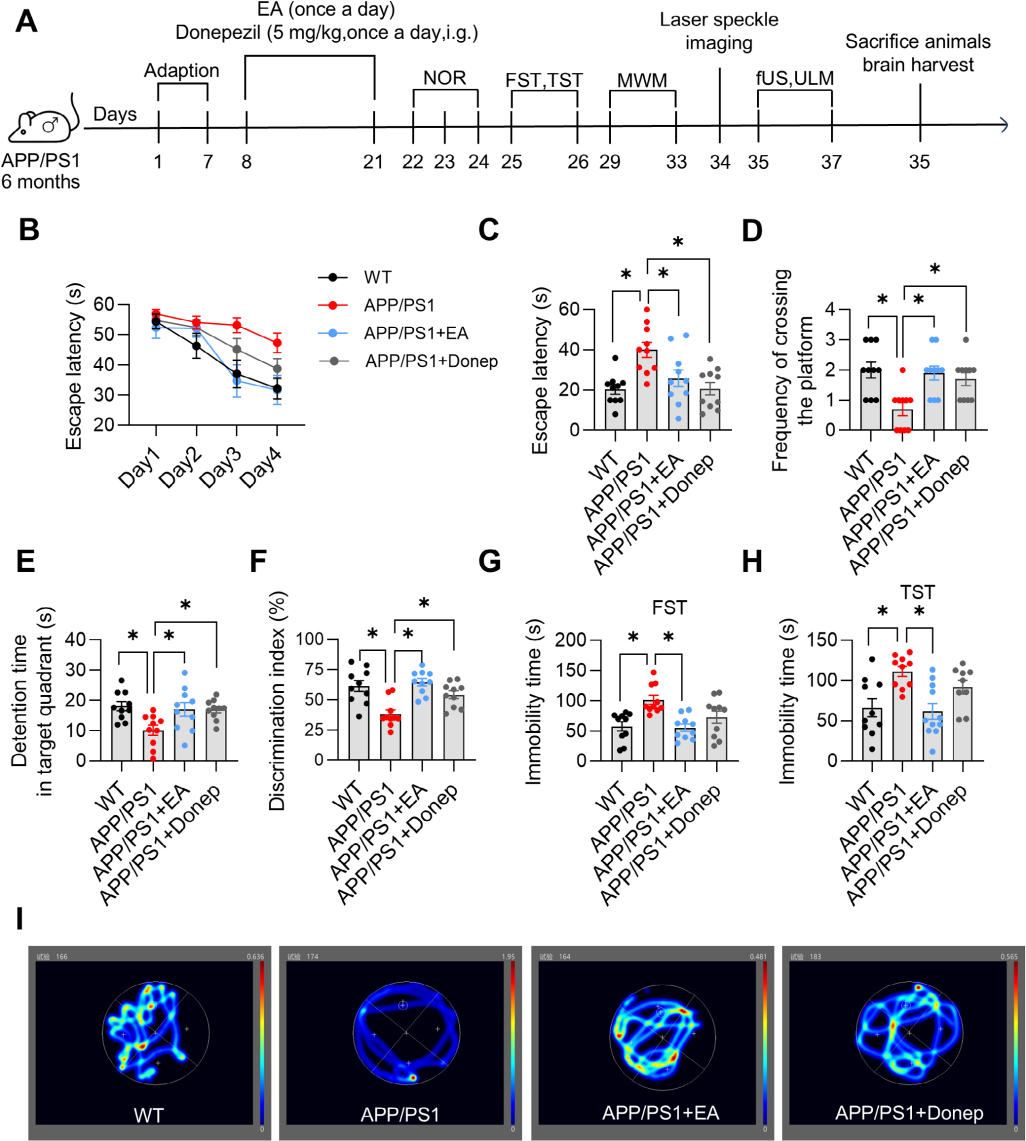
Electroacupuncture ameliorates AD‐like phenotypes in APP/PS1 mice. (A) Experimental flowchart for *Experiment 2*. (B) Escape latencies during the spatial navigation trial. (C) The escape latency during the spatial probe trial. (D) Frequency of crossing the platform in the target quadrant during the spatial probe trial. (E) Time spent in the target quadrant during the spatial probe trial. (F) Discrimination index in the new object recognition test. (G) Immobility time in the forced swim test. (H) Immobility time in the tail suspension test. (I) Representative traces during the spatial probe trial. Data are expressed as mean ± SEM (*n* = 10 per group). *p < 0.05 between groups. (one‐way ANOVA followed by *Tukey* test).

Next, we evaluated the effect of EA on AD‐like phenotypes in 6‐month‐old APP/PS1 (hereafter referred to as APP/PS1) mice (Figure 3A). The results of MWM showed that the escape latencies of all groups were gradually shortened during the spatial navigation trial (Figure 3B). During the spatial exploration trial, APP/PS1 mice displayed significantly prolonged escape latency (*p* < 0.05, Figure 3C), reduced frequency of crossing the platform (*p* < 0.05, Figure 3D), and decreased detention time in the target quadrant (*p* < 0.05, Figure 3E) compared to age‐matched WT mice. Both APP/PS1 + EA and APP/PS1 + Donep groups showed significant improvements relative to APP/PS1 mice, including shortened escape latency (*p* < 0.05, *F* (3, 36) = 7.167, Figure 3C), frequency of crossing the platform (*p* < 0.05, *F* (3, 36) = 6.707, Figure 3D), and increased detention time in the target quadrant (*p* < 0.05, *F* (3, 36) = 4.668, Figure 3E). Representative traces during the spatial exploration trial were shown in Figure 3I. The NOR test revealed significantly decreased discrimination index in APP/PS1 mice compared to WT mice, while both APP/PS1 + EA and APP/PS1 + Donep groups exhibited significantly increased discrimination index relative to APP/PS1 mice (*p* < 0.05, *F* (3, 36) = 10.41, Figure 3F). In FST and TST, APP/PS1 mice demonstrated significantly prolonged immobility time compared to age‐matched WT mice. The APP/PS1 + EA group showed significantly reduced immobility time in both FST (*p* < 0.05, *F* (3, 36) = 7.071, Figure 3G) and TST (*p* < 0.05, *F* (3, 36) = 6.492, Figure 3H) compared to APP/PS1 mice, whereas the APP/PS1 + Donep group showed no significant differences (*p* > 0.05; Figure 3G,H). These results demonstrate that EA intervention ameliorates AD‐like phenotypes in APP/PS1 mice.

### 3.4 EA Improves CBF and Microvascular Ultrastructure in APP/PS1 Mice

**FIGURE 4:**
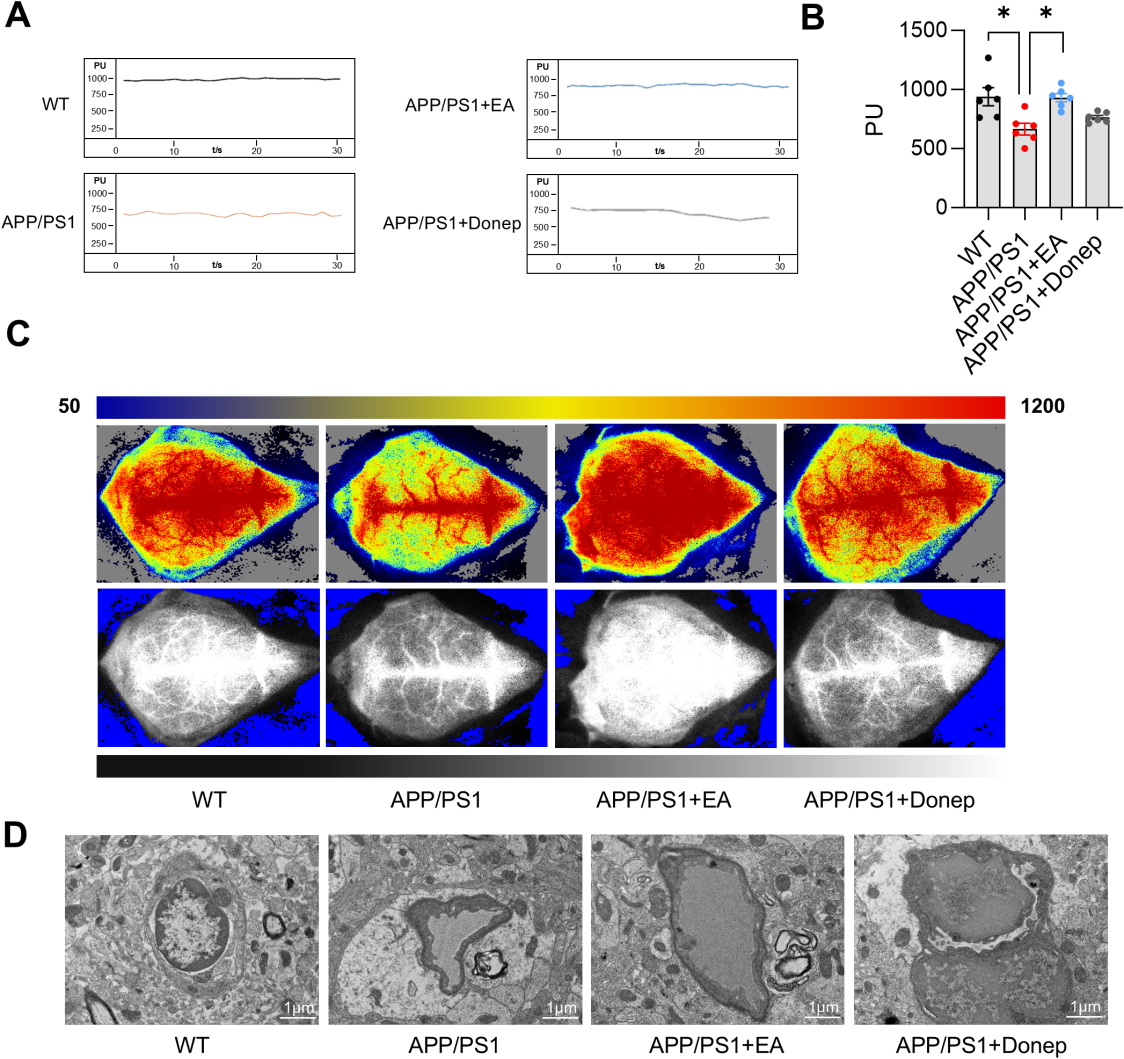
Electroacupuncture improves cerebral blood flow and microvascular ultrastructure in APP/PS1 mice. (A) Representative images of cerebral blood flow changes in the cortex measured by laser Doppler flowmetry. (B) Quantitative data of cerebral blood flow changes in the cortex. (C) Representative images of cerebral blood flow changes in the cortex observed by laser speckle imaging. (D) Representative images of microvascular ultrastructure in mice (5000 x). Scale bar: 1 μm. Data are expressed as mean ± SEM (*n* = 6 per group). *p < 0.05 between groups (one‐way ANOVA followed by *Tukey* test).

Laser speckle imaging and laser Doppler blood flowmetry revealed significantly reduced CBF in APP/PS1 mice compared to age‐matched WT mice. By contrast, EA treatment, but not Donep, effectively restored cerebral perfusion in APP/PS1 mice (*p* < 0.05, *F* (3, 20) = 7.273, Figure 4A–C). These results indicate that EA intervention inhibits the reduction of CBF in APP/PS1 mice. Observation of microvascular ultrastructure by TEM revealed severe morphological shrinkage in APP/PS1 mice compared with the WT mice. EA intervention markedly ameliorated these abnormalities, while donepezil treatment did not produce significant improvement in microvascular degeneration (Figure 4D).

### 3.5 EA Prevents Synaptic Defects in the Hippocampus of APP/PS1 Mice

**FIGURE 5:**
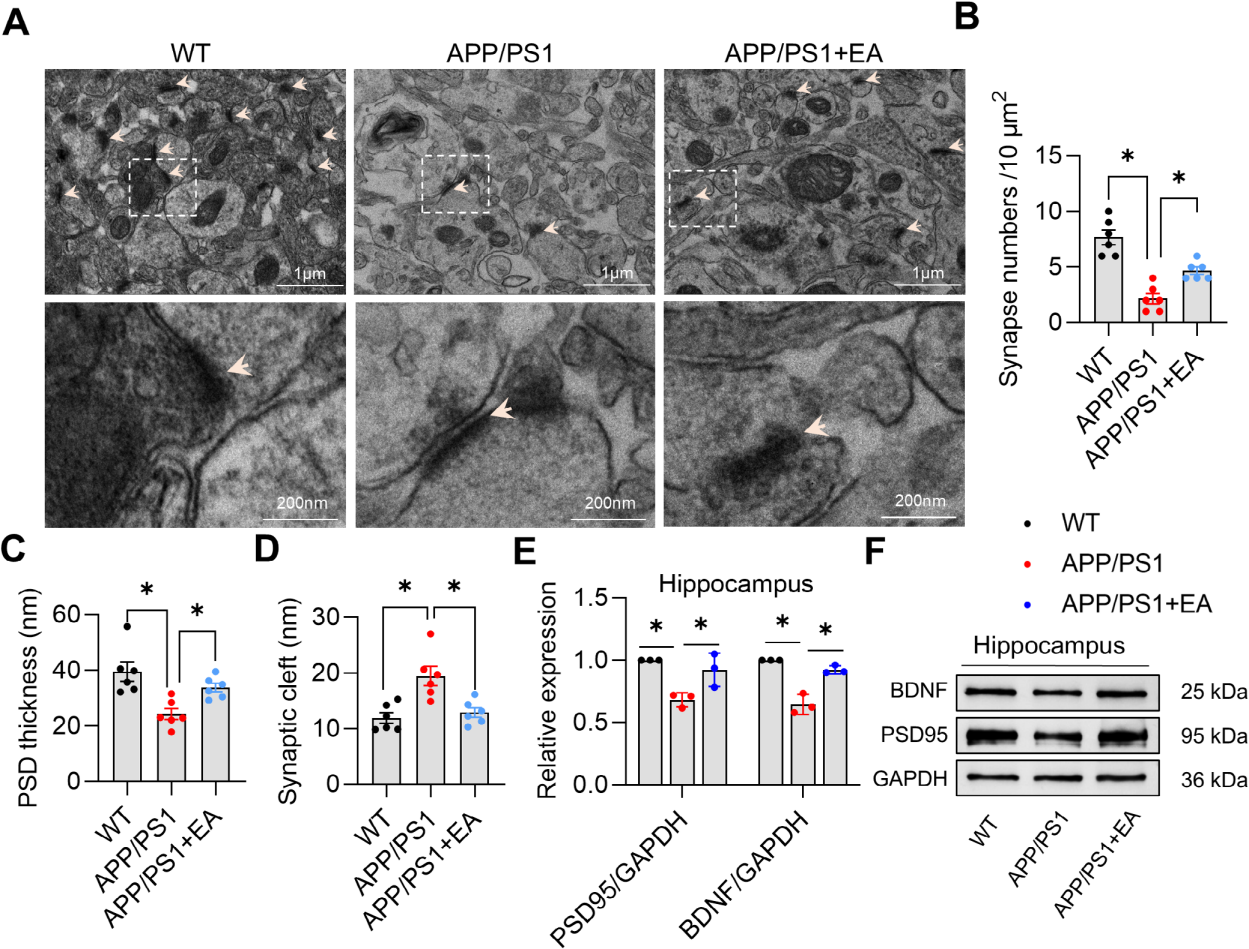
Electroacupuncture prevents synaptic defects in the hippocampus of APP/PS1 mice. (A) Representative images of synaptic ultrastructure in the hippocampus observed by transmission electron microscope (5000 x). Scale bar: 1 μm. (B) Quantitative analysis of synapse numbers. (C) Quantitative analysis of PSD thickness. (D) Quantitative analysis of synaptic cleft. (E) Quantitative analysis of PSD95 and BDNF expression in the hippocampus of mice. (F) Representative immunoblots of PSD95 and BDNF expression. Data are expressed as mean ± SEM (*n* = 6 or 3 per group). *p < 0.05 between groups. (one‐way ANOVA followed by *Tukey* test).

TEM analysis revealed that APP/PS1 mice exhibited synaptic ultrastructural impairments characterized by reduced synaptic vesicles, disorganized architecture, and decreased synapse numbers compared to WT mice. Following EA treatment, these deficits were significantly ameliorated, including increased synapse numbers (*p* < 0.05, *F* (2, 15) = 29.04, Figure 5A,B), PSD thickness (*p* < 0.05, *F* (2, 15) = 9.409, Figure 5A,C), and synaptic clefts (*p* < 0.05, *F* (2, 15) = 10.77, Figure 5A,D). Consistent with these findings, western blot analysis showed significant decreased expression of BDNF (*p* < 0.05, *F* (2, 6) = 41.29, Figure 5E,F) and PSD95 (*p* < 0.05, *F* (2, 6) = 11.59, Figure 5E,F) in APP/PS1 mice, which was effectively reversed by EA intervention. Although neuronal pathology in AD models manifests later than synaptic alterations [19], our findings demonstrate that electroacupuncture effectively mitigates hippocampal neuronal degeneration (Figure S1). Collectively, these data demonstrate that EA intervention effectively attenuates synaptic degeneration in APP/PS1 mice.

### 3.6 EA Inhibits Abnormal Expression of Microvessels and Inflammation‐Related Proteins in APP/PS1 Mice

**FIGURE 6:**
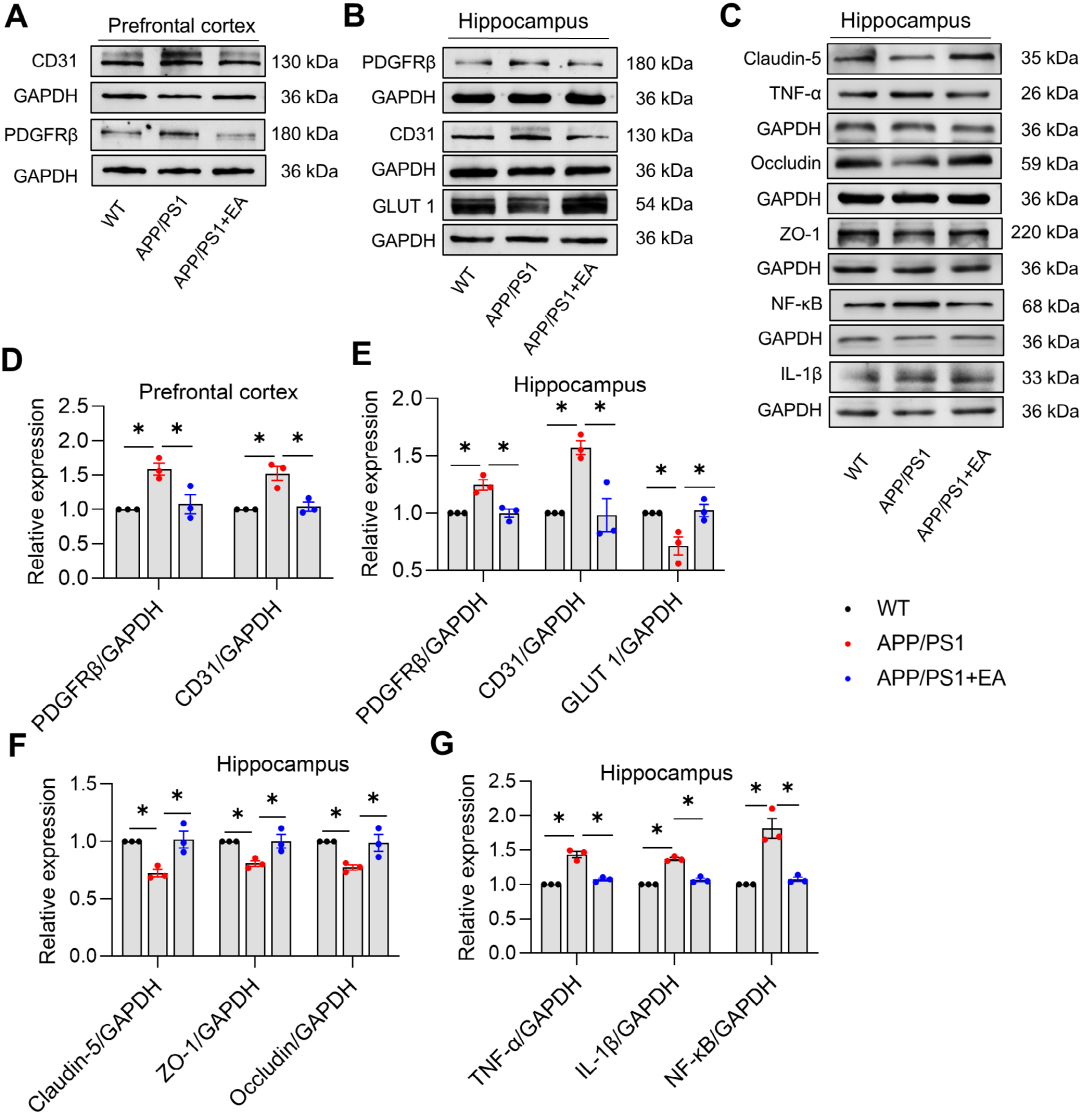
Electroacupuncture inhibits abnormal expression of microvessels and inflammation‐related proteins in APP/PS1 mice. (A) Representative immunoblots of PDGFRβ and CD31 expression in the prefrontal cortex. (B) Representative immunoblots of PDGFRβ, CD31, and GLUT1 expression in the hippocampus. (C) Representative immunoblots of Occludin, Claudin‐5, ZO‐1, NF‐κB, TNF‐α, and IL‐1β expression in the hippocampus. (D) Quantitative data of PDGFRβ and CD31 expression in the prefrontal cortex. (E) Quantitative data of PDGFRβ, CD31, and GLUT1 expression in the hippocampus. (F) Quantitative analysis of Occludin, Claudin‐5, and ZO‐1 expression in the hippocampus. (G) Quantitative analysis of NF‐κB, TNF‐α, and IL‐1β expression in the hippocampus. Data are expressed as mean ± SEM (*n* = 3 per group). *p < 0.05 between groups. (one‐way ANOVA followed by *Tukey* test).

Western blot analysis revealed that APP/PS1 mice exhibited significantly increased expression of the pericyte marker PDGFRβ (prefrontal cortex: *p* < 0.05, *F* (2, 6) = 11.52, Figure 6A,D; hippocampus: *p* < 0.05, *F* (2, 6) = 18.04, Figure 6B,E, respectively) and endothelial marker CD31 (prefrontal cortex: *p* < 0.05, *F* (2, 6) = 16.76, Figure 6A,D; hippocampus: *p* < 0.05, *F* (2, 6) = 13.80, Figure 6B,E, respectively) in both the hippocampus and prefrontal cortex compared to WT mice. Specific analysis of hippocampal tissue further showed marked reductions in GLUT1 (*p* < 0.05, *F* (2, 6) = 9.495, Figure 6B,E), Occludin (*p* < 0.05, *F* (2, 6) = 8.286, Figure 6C,F), Claudin 5 (*p* < 0.05, *F* (2, 6) = 12.34, Figure 6C,F), and ZO‐1 (*p* < 0.05, *F* (2, 6) = 9.032, Figure 6C,F), along with elevated NF‐κB (*p* < 0.05, *F* (2, 6) = 27.95, Figure 6C,G), TNF‐α (*p* < 0.05, *F* (2, 6) = 62.26, Figure 6C,G), and IL‐1β (*p* < 0.05, *F* (2, 6) = 102.1, Figure 6C,G) expression. Importantly, EA intervention effectively reversed all these molecular abnormalities. Together, these results demonstrate that EA ameliorates hippocampal microangiopathy and neuroinflammation in APP/PS1 mice.

### 3.7 Sunitinib Ameliorates Cognitive Decline and Cerebral Microangiopathy in APP/PS1 Mice

**FIGURE 7:**
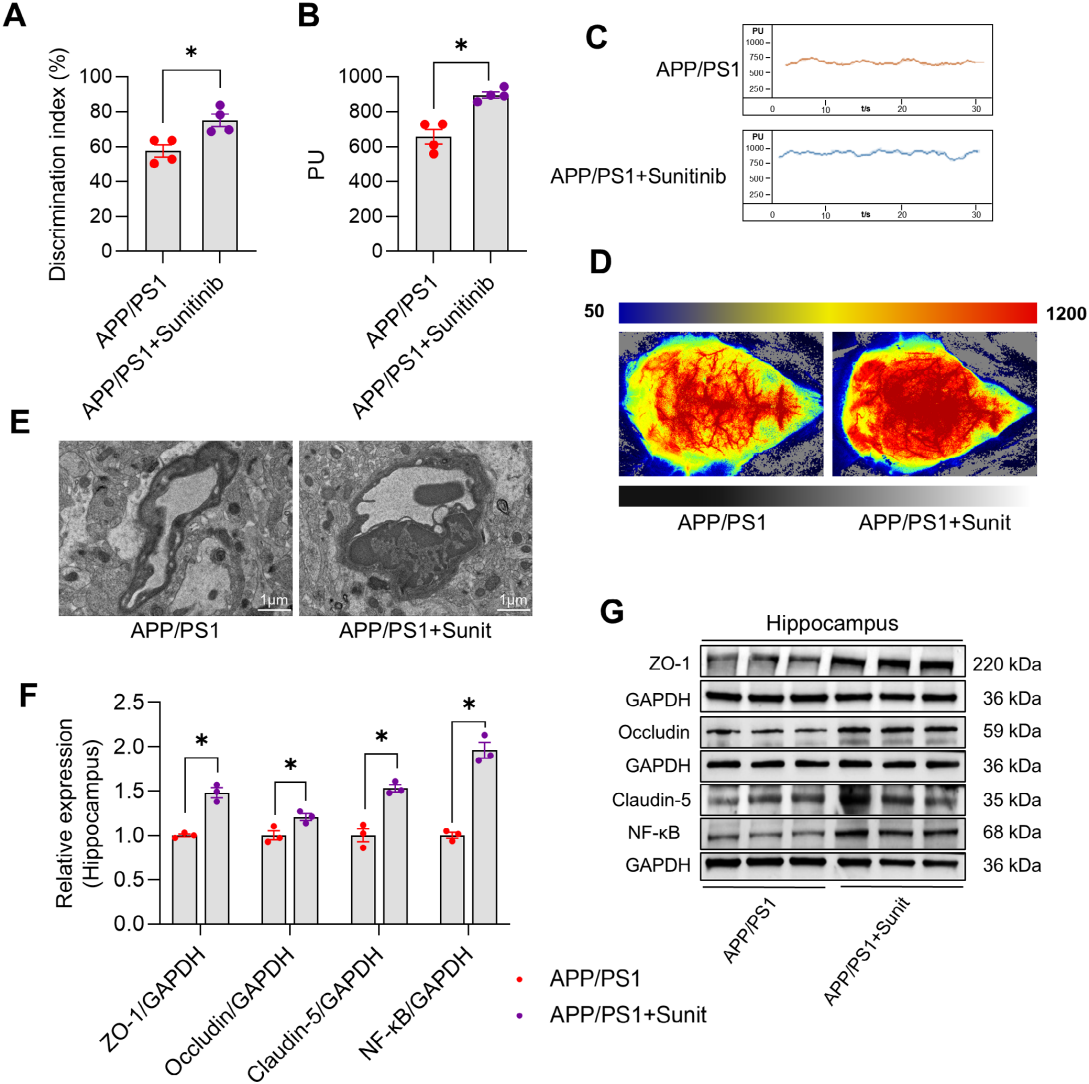
Sunitinib ameliorates cognitive decline and cerebral microangiopathy in APP/PS1 mice. (A) Discrimination index in the new object recognition test. (B) Quantitative data of cerebral blood flow changes in the cortex measured by laser Doppler flowmetry. (C) Representative images of cerebral blood flow changes in the cortex measured by laser Doppler flowmetry. (D) Representative images of cerebral blood flow changes in the cortex observed by laser speckle imaging. (E) Representative images of hippocampal microvasculature observed by transmission electron microscope (5000 x). Scale bar: 1 μm. (F) Representative immunoblots of Occludin, Claudin‐5, ZO‐1, and NF‐κB expression in the hippocampus. (G) Quantitative analysis of Occludin, Claudin‐5, ZO‐1, and NF‐κB expression in the hippocampus. Data are expressed as mean ± SEM (*n* = 4 or 3 per group). *p < 0.05 between groups. (Independent student *t*‐test).

We further evaluated the effects of vascular regulation on the AD‐like phenotype of APP/PS1 mice using PDGFRβ inhibitor. The NOR test demonstrated a significantly increased discrimination index in APP/PS1 + Sunitinib mice compared to APP/PS1 mice (*p* < 0.05, *t* = 3.477, df = 6, Figure 7A). Laser doppler blood flowmetry confirmed significantly higher perfusion in APP/PS1 + Sunitinib mice versus APP/PS1 mice (*p* < 0.05, *t* = 5.165, df = 6, Figure 7B,C). Laser speckle imaging showed increased CBF in APP/PS1 + Sunitinib mice (Figure 7D). TEM revealed hippocampal microvascular constriction, luminal stenosis, and perivascular edema in APP/PS1 mice, all of which were reversed following Sunitinib treatment (Figure 7E). Western blot analysis further confirmed that Sunitinib treatment significantly elevated the expression of hippocampal Occludin (*p* < 0.05, *t* = 3.140, df = 4, Figure 7F,G), Claudin‐5 (*p* < 0.05, *t* = 6.272, df = 4, Figure 7F,G), and ZO‐1 (*p* < 0.05, *t* = 8.326, df = 4, Figure 7F,G) in APP/PS1 mice. In addition, Sunitinib also significantly increased NF‐κB expression in APP/PS1 mice (*p* < 0.05, *t* = 10.21, df = 4, Figure 7F,G). Collectively, these data indicate that Sunitinib ameliorates CBF reduction and micropathology, and improves AD‐like behaviors.

### 3.8 EA Enhances the Functional Connection Between Hippocampal Regions in APP/PS1 Mice

**FIGURE 8:**
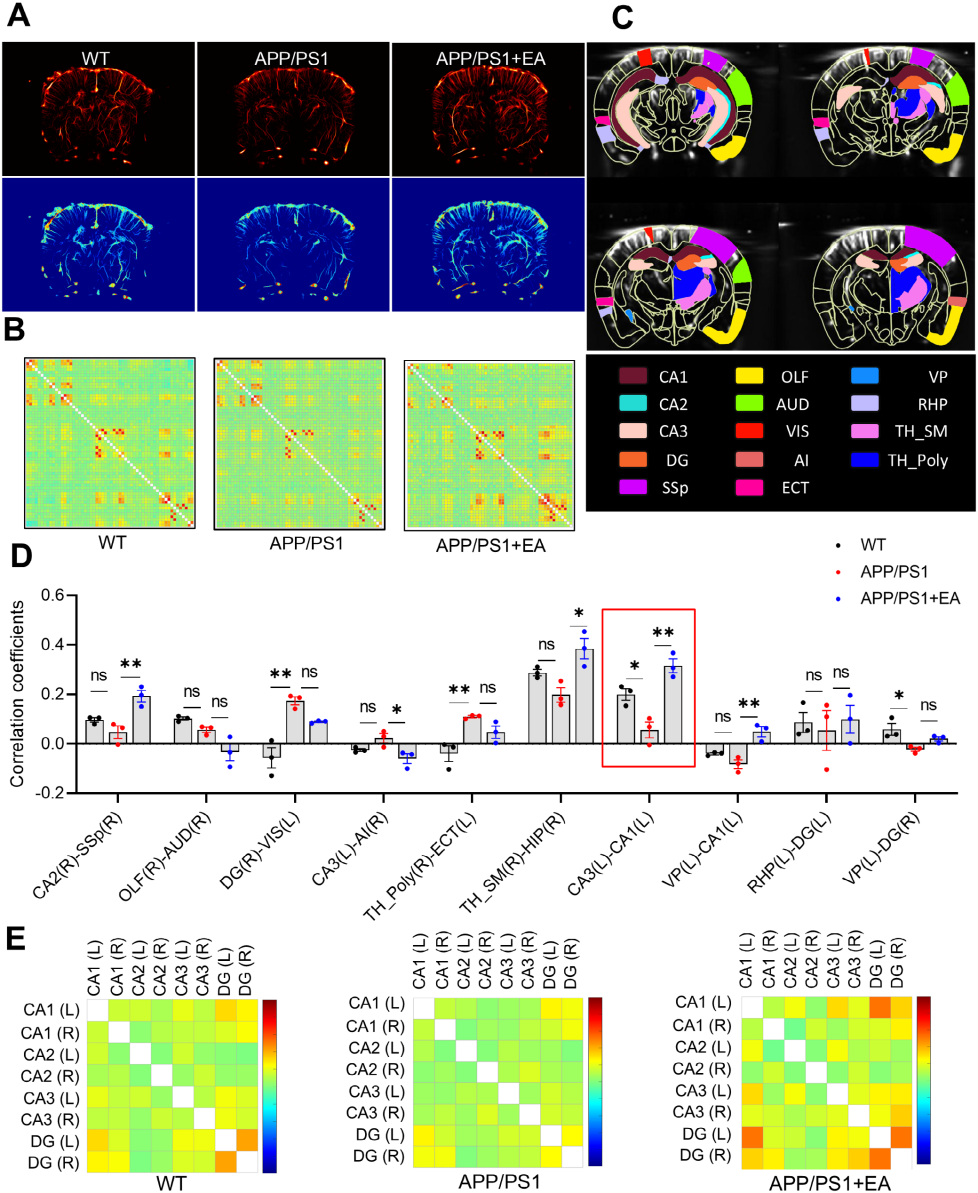
EA enhances the functional connection between hippocampal regions in APP/PS1 mice. (A) Representative image of blood flow direction in mice. (B) Comprehensive functional connectivity mapping across 106 brain regions. (C) Volumetric scanning of each sampling unit (corresponding to 4 slices) along a 1.6 mm axial dimension within 2.2 s acquisition time. (D) Quantitative analysis of functional connectivity in different brain regions of mice. (E) Functional connectivity strength between hippocampal subregions (group mean values shown). Data are expressed as mean ± SEM (*n* = 3 per group). *p < 0.05 between groups. (one‐way ANOVA followed by *Tukey* test).

Representative images of CBF direction using ULM are shown in Figure 8A. Comparative analysis of functional connectivity among the WT, APP/PS1, and APP/PS1 + EA groups identified statistically significant connections using MATLAB. “TRUE” data point was defined as one exhibiting a significant difference in at least two of the three group comparisons (Figure S2). Whole‐brain functional connectivity mapping across 106 regions revealed partially disrupted networks in APP/PS1 mice compared to WT mice, which were substantially restored following EA intervention (Figure 8B–E). Among the top 10 most significant connections, 20 key brain regions were implicated, including 10 hippocampal and 5 cortical subregions. Hippocampal regions comprised Field CA1 (CA1), Field CA2 (CA2), Field CA3 (CA3), dentate gyrus (DG), hippocampal region (HIP), and retrohippocampal region (RHP), while cortical areas included the primary somatosensory area (SSp), olfactory areas (OLF), auditory areas (AUD), visual areas (VIS), agranular insular area (AI), and ectorhinal area (ECT). Statistical comparisons between WT and APP/PS1 mice showed significantly altered functional connectivity in the following region pairs: DG(R)–VIS(L), thalamus polymodal association cortex related (TH_Poly) (R)–ECT(L), CA3(L)–CA1(L), and pallidum ventral region (VP) (L)–DG(R). EA specifically modulated connectivity in CA2(R)–SSp(R), CA3(L)–AI(R), thalamus sensory motor cortex related (TH_SM) (R)–HIP(R), CA3(L)–CA1(L), and VP(L)–CA1(L) relative to the APP/PS1 mice (Figure 8D). The average functional connectivity strength among hippocampal subregions across experimental groups was presented as a heatmap (Figure 8E). Together, these findings indicate that the hippocampus and cortex represent key brain regions for intervening in AD‐like phenotypes, and EA mainly enhances the functional connection between hippocampal CA1 and CA3 regions in APP/PS1 mice.

## 4 Discussion

In this study, we found that EA could improve AD‐like phenotypes, including cognitive impairment and pathological changes in neurons and synapses. Most importantly, EA improved cerebral microangiopathy in APP/PS1 mice. This study provides an important experimental basis for the application of EA in the treatment of AD.

### 4.1 Cerebral Microangiopathy May Precede Cognitive Decline

Cerebral microangiopathy is now recognized as one of the major contributors to age‐related cognitive impairment and dementia [19, 20, 21]. Although the underlying mechanisms remain unclear, the vascular hypothesis has been repeatedly validated in numerous community‐based clinicopathological studies. Notably, a recent human cerebrovascular atlas study revealed that 30 out of the top 45 AD genome‐wide association study (GWAS) genes were expressed in cerebrovascular regions [22]. Cerebral microangiopathy‐induced hypoperfusion may precede the accumulation of Aβ and p‐Tau, serving as a precursor for the later development of AD and AD‐related dementia (ADRD) [7, 23]. Potential therapies targeting cerebral perfusion have shown promise in enhancing cognitive function in AD patients, suggesting that the mechanisms underlying cerebral hypoperfusion may involve impaired vascular autoregulation and neurovascular coupling [23, 24]. Importantly, researchers have classified adults along the AD continuum into three groups: cognitively unimpaired (CU) Aβ‐negative (−), CU Aβ‐positive (+), and cognitively impaired (CI) Aβ+. When measuring CBF, it was found that in CU participants, lower CBF was associated with altered biomarkers of Aβ, tau, and synaptic dysfunction, highlighting that CBF reduction occurs early in the AD continuum [25]. Our previous study demonstrated that Aβ induces excessive interaction between cerebral pericytes and endothelial cells, leading to cerebral microangiopathy and CBF reduction [15]. Importantly, quantitative proteomic analysis of bulk brain tissue and isolated cerebral vasculature from the same individuals revealed that protein products of AD risk gene loci are predominantly concentrated within cerebrovascular modules [26]. These reports collectively suggest that cerebral microangiopathy may play a significant role in the initiation and progression of AD.

To rule out the potential effect of gender as a confounding variable in the pathogenesis of AD [13], female animals were not selected for this study, but this does have limitations. Our results demonstrated that compared to age‐matched WT mice, 6‐month‐old and 9‐month‐old APP/PS1 mice showed cognitive decline, while 3‐month‐old APP/PS1 mice did not exhibit cognitive impairment. Interestingly, CBF was reduced and cerebral microvascular markers increased in 3‐, 6‐, and 9‐month‐old APP/PS1 mice compared to their age‐matched WT mice. These findings indicate that cerebral microvascular dysfunction occurs prior to cognitive impairment, likely representing a core pathological feature of early AD that initiates a cascade of events, including blood–brain barrier (BBB) disruption, decreased CBF, neuroinflammatory responses, and synaptic dysfunction, ultimately leading to neurodegeneration. Timely intervention at this stage could effectively prevent later‐stage symptoms such as cognitive impairment and dementia.

### 4.2 EA At Acupoints GV20, CV4, and ST36 Can Prevent the Occurrence of AD‐Like Phenotypes

Clinical studies have shown that acupuncture has good clinical efficacy in AD patients [27, 28]. Under the guidance of TCM theory, this study selected a combination of three acupoints (GV20, CV4, and ST36) for intervention. Among them, GV20 is the intersection point between bladder meridian and Governor Vessel, which can regulate and replenish the central qi, strengthen the brain and calm the mind; CV4 is the foundation of vital energy, which is good at warming and replenishing the innate vital energy, and indirectly nourishing the heart and calming the mind by cultivating the essence and strengthening the foundation; ST36 can effectively regulate qi and blood circulation. The combination of the above acupoints can not only adjust the mind and strengthen the body but also achieve the effect of treating AD.

Our results showed that EA at acupoints GV20, CV4, and ST36 can improve AD‐like behaviors and cerebral microvascular dysfunction in APP/PS1 mice. In fact, as a central brain region for learning memory and emotional regulation, the hippocampus exhibits special susceptibility to ischemia, hypoxia and neuroinflammation due to its high metabolic requirement [29]. Aβ can spread to the whole brain during ad [30], in which the hippocampus region exhibits special vulnerability, and its structural and functional impairment is closely related to cognitive decline and emotional abnormalities [31, 32]. Our results also confirmed that APP/PS1 mice exhibited AD‐like phenotypes including neuroinflammation and synaptic deficits, which were highly consistent with early symptoms of clinical AD patients [33]. It is worth noting that we also found that APP/PS1 mice exhibited significant functional connectivity deficits between the hippocampus and multiple brain regions, suggesting that this may be related to the neuropathological mechanisms of AD. EA can suppress inflammatory response and synaptic deficits, and especially enhance functional connections in the hippocampal regions of APP/PS1 mice. Collectively, EA at acupoints GV20, CV4, and ST36 can prevent the occurrence of AD‐like phenotypes.

### 4.3 Inhibition of Cerebral Microangiopathy to Improve CBF May Be the Biological Mechanism of EA

In the early stages of AD progression, basal CBF is reduced, and cerebral microvascular networks are impaired [34]. The reason may be that oligomer Aβ causes pericytes to contract, which leads to capillary dysfunction and, ultimately, insufficient CBF perfusion and brain microcirculation disorders [35]. Inadequate brain perfusion can directly lead to degradation of tight junction proteins, destroying the structural integrity of the BBB [36]. Abnormal reduction of CBF, leading to synaptic injury, is associated with worsening of neurological function [37]. Therefore, the timely CBF recovery would prevent the later change of synaptic injury or neuronal death [38]. Our previous study indeed supported that reduced CBF due to cerebral microangiopathy was responsible for the cognitive decline in Aβ‐infused mice [15].

EA has demonstrated multi‐target regulatory advantages in ameliorating cerebral microcirculatory disturbances [39]. To ensure the validity of EA intervention, our preliminary experiments showed that mice in the anesthetized group had no changes in cognitive function and CBF. Building upon this verification, our present results revealed that APP/PS1 mice exhibited pathological alterations at a low age, including reduced CBF, aberrant expression of cerebral microvascular‐related molecules, and damaged microvascular structure. EA treatment prevented the decline in CBF and promoted the expression of microvascular markers such as PDGFRβ and CD31 in both the hippocampus and cortex, while concurrently upregulating hippocampal GLUT1 expression in APP/PS1 mice. Based on these data, EA was found to increase CBF in AD model mice and enhance the expression of GLUT1. These results suggest that EA intervention may improve the supply of oxygen and nutrients to neurons, thereby creating favorable conditions for the restoration of synaptic function. In reality, the neurovascular unit (NVU) operates as an integrated functional system, and vascular impairment can lead to dysfunction of the entire NVU, thereby laying the pathological foundation for cognitive deficits [19]. Although existing theories support this view, the direct relationship between changes in CBF and cognitive impairment still requires further investigation.

We have previously reported that sunitinib, as an inhibitor of PDGFRβ in cerebral microvascular pericytes, can improve cognitive function in mice injected with Aβ [15]. This experimental design was used to verify the potential mechanism of EA. Our results showed that sunitinib intervention effectively improved CBF reduction and prevented cerebral microvascular structure damage in APP/PS1 mice. Collectively, these findings suggest that EA may ameliorate AD progression by modulating hyperactive pericyte contractility, thereby preserving cerebral perfusion and exerting early preventive effects. Furthermore, our results demonstrated that EA intervention effectively attenuated elevated inflammatory responses in the hippocampus of APP/PS1 mice. Substantial evidence has established neuroinflammation as a critical pathological component in AD brains [40, 41]. Notably, previous studies have confirmed that ischemic conditions indirectly activate microglia and astrocytes, leading to the production of pro‐inflammatory cytokines [42, 43, 44], which is consistent with our experimental observations. Surprisingly, in contrast to the EA effect, sunitinib significantly promoted the expression of NF‐κB in APP/PS1 mice. This suggests that although sunitinib may improve cognitive function by preventing cerebral microangiopathy, it also risks inducing inflammatory responses, which also highlights that using EA treatment is relatively safer. In summary, these data indicate that EA may confer therapeutic benefits in AD through mechanisms involving improvement of cerebral microcirculation and suppression of neuroinflammatory responses.

### 4.4 Limitations of This Study

EA exerts beneficial effects on cognitive impairment across various diseases through either shared or distinct mechanisms. However, further investigation is required to elucidate the specific mechanisms underlying the effects of different acupoint combinations. Moreover, growing evidence identifies gut microbiota and their metabolites as key contributors to cognitive impairment pathogenesis. The gut‐brain axis may represent a promising therapeutic target for acupuncture in AD treatment. Future studies should particularly focus on delineating how EA specifically modulates microvascular pericyte function to mediate its therapeutic effects.

## 5 Conclusion

**FIGURE 9:**
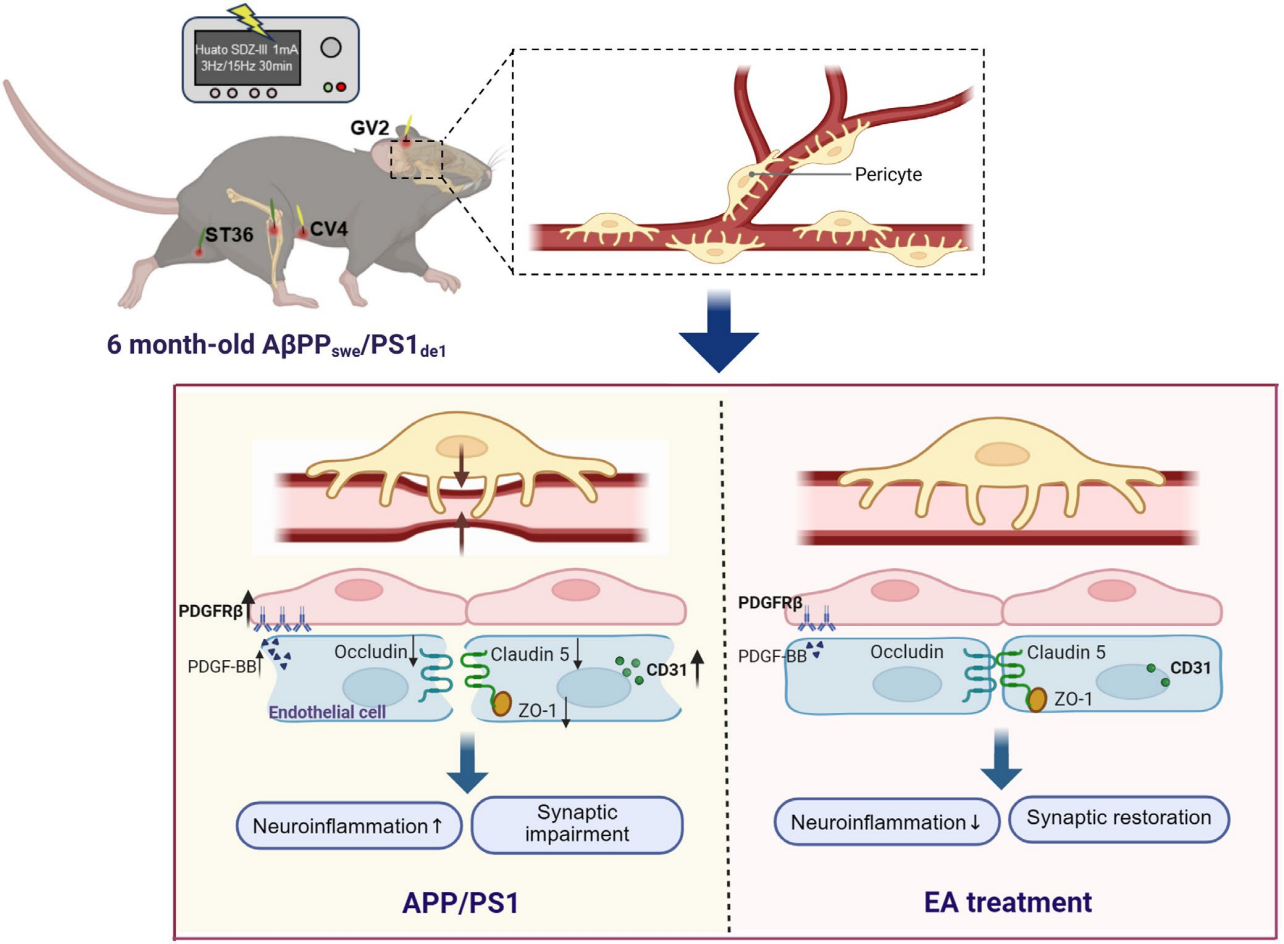
Schematic illustration of the potential mechanisms of electroacupuncture against AD‐like phenotypes. Electroacupuncture at combined acupoints CV4, ST36, and GV20 may ameliorate AD‐like phenotypes in APP/PS1 mice, including hippocampal pathological damage, neuroinflammation, and cognitive decline, potentially through preventing cerebral microvascular pathology and increasing cerebral blood flow.

Our study demonstrates that EA at combined acupoints GV20, CV4, and ST36 ameliorates AD‐like phenotypes, potentially through preventing microangiopathy and enhancing CBF (Figure 9). These findings provide novel insights for early intervention strategies to halt cognitive decline in AD.

## Author Contributions

Conceptualization, C.Y.; methodology, B.L.; validation, B.L.; resources, S.Y.; data curation, C.Y.; writing‐original draft preparation, C.Y.; writing‐review and editing, J.w., X.W., and G.Z.; project administration, J.w., X.W., and G.Z.; funding acquisition, J.w., X.W., and G.Z. All authors have read and agreed to the published version of the manuscript.

## Funding

This research was supported by the Research Funds of the Center for Xin'an Medicine and Modernization of Traditional Chinese Medicine of IHM (Nos. 2023CXMMTCM013 and 2023CXMMTCM021), the National Natural Science Foundation of China (No. 82474633), the Anhui Provincial Key R&D Programme (No. 202304295107020105), the Anhui Natural Science Foundation (Nos. 2508085MH226, 2208085MH282), the Key Project of Anhui Natural Science Research (No. 2024AH051045), and the Open Fund for Key Disciplines of Basic Theory of Traditional Chinese Medicine (No. ZYJCLLYB‐11).

## Disclosure

*Institutional Review Board Statement*: The animal study protocol was approved by the Experimental Animal Ethics Committee of Anhui University of Chinese Medicine (Hefei, Anhui, China) (protocol code AHUCM‐mouse‐2023146 and date of approval 10 October 2024).

## Consent

The authors have nothing to report.

## Conflicts of Interest

The authors declare no conflicts of interest.

## Supporting information


**Figure S1:** Electroacupuncture prevents hippocampal neuronal degeneration in APP/PS1 mice. (A) Representative images of hippocampal CA1 and DG regions measured by HE staining (200 x). Scale bar: 50 μm. (B) Representative images of hippocampal CA1 and DG regions measured by Nissl staining (200 x). Scale bar: 50 μm. (C) Illustration of a mouse brain section. Nissl staining was observed in the region depicted by the rectangular box. (D‐E) The mean optical density of Nissl bodies of hippocampal CA1 and DG regions. Data are expressed as mean ± SEM (*n* = 3 per group). *p < 0.05 between groups. (one‐way ANOVA followed by *Tukey* test).
**Figure S2:** Binary matrix of significant functional connectivity derived from MATLAB analysis.

## Data Availability

All the data is contained within the article, and the data presented in this study are available upon request from the corresponding author.
