# Supplementary material for: Electroacupuncture Prevents Against AD‐Like Phenotypes in APP/PS1 Mice: Investigation of the Mechanisms From Cerebral Microangiopathy
Source: CNS Neurosci Ther. 2025 Dec 9;31(12):e70696. doi: 10.1002/cns.70696 (PMC12689941; doi:10.1002/cns.70696)
Supplement: Supplementary file 1 — Figure S1: Electroacupuncture prevents hippocampal neuronal degeneration in APP/PS1 mice. (A) Representative images of hippocampal CA1 and DG regions measured by HE staining (200 x). Scale bar: 50 μm. (B) Representative images of hippocampal CA1 and DG regions measured by Nissl staining (200 x). Scale bar: 50 μm. (C) Illustration of a mouse brain section. Nissl staining was observed in the region depicted by the rectangular box. (D‐E) The mean optical density of Nissl bodies of hippocampal CA1 and DG regions. Data are expressed as mean ± SEM (n = 3 per group). *p < 0.05 between groups. (one‐way ANOVA followed by Tukey test). Figure S2: Binary matrix of significant functional connectivity derived from MATLAB analysis. [file CNS-31-e70696-s001.docx]

**Electroacupuncture prevents against AD-like phenotypes in APP/PS1 mice: Investigation of the mechanisms from cerebral microangiopathy**

Chen Yang^a,b^, Baobao Li^a,b^, Shaojie Yang ^a,c^, Xuncui Wang^a,b,*^, Guoqi Zhu^a,b,*^, Jingji wang ^a,c,*^

(*^a^* *Center for Xin’an Medicine and Modernization of Traditional Chinese Medicine of IHM, and Key Laboratory of Molecular Biology (Brain diseases), Anhui University of Chinese Medicine, Hefei 230012, China;*

*^b^ Key Laboratory of Xin’an Medicine, the Ministry of Education, Hefei 230038, China;*

*^c^ The Second Affiliated Hospital of Anhui University of Chinese Medicine, Hefei 230061, China.*

***Corresponding authors:**

Xuncui Wang (X. Wang),

Anhui University of Chinese Medicine, 103 Meishan Road, Hefei 230038, China.

E-mail: [wangxuncui@163.com](mailto:wangxuncui@163.com). Tel: 86551-68129793

Guoqi Zhu (G. Zhu),

Anhui University of Chinese Medicine, 350 Longzihu Road, Hefei 230012, China.

E-mail: [guoqizhu@gmail.com](mailto:guoqizhu@gmail.com). Tel: 86551-68129028

Jingji wang (J. Wang),

The Second Affiliated Hospital of Anhui University of Chinese Medicine, 300 Shouchun Road, Hefei 230061, China.

E-mail: wjjglacial@163.com.


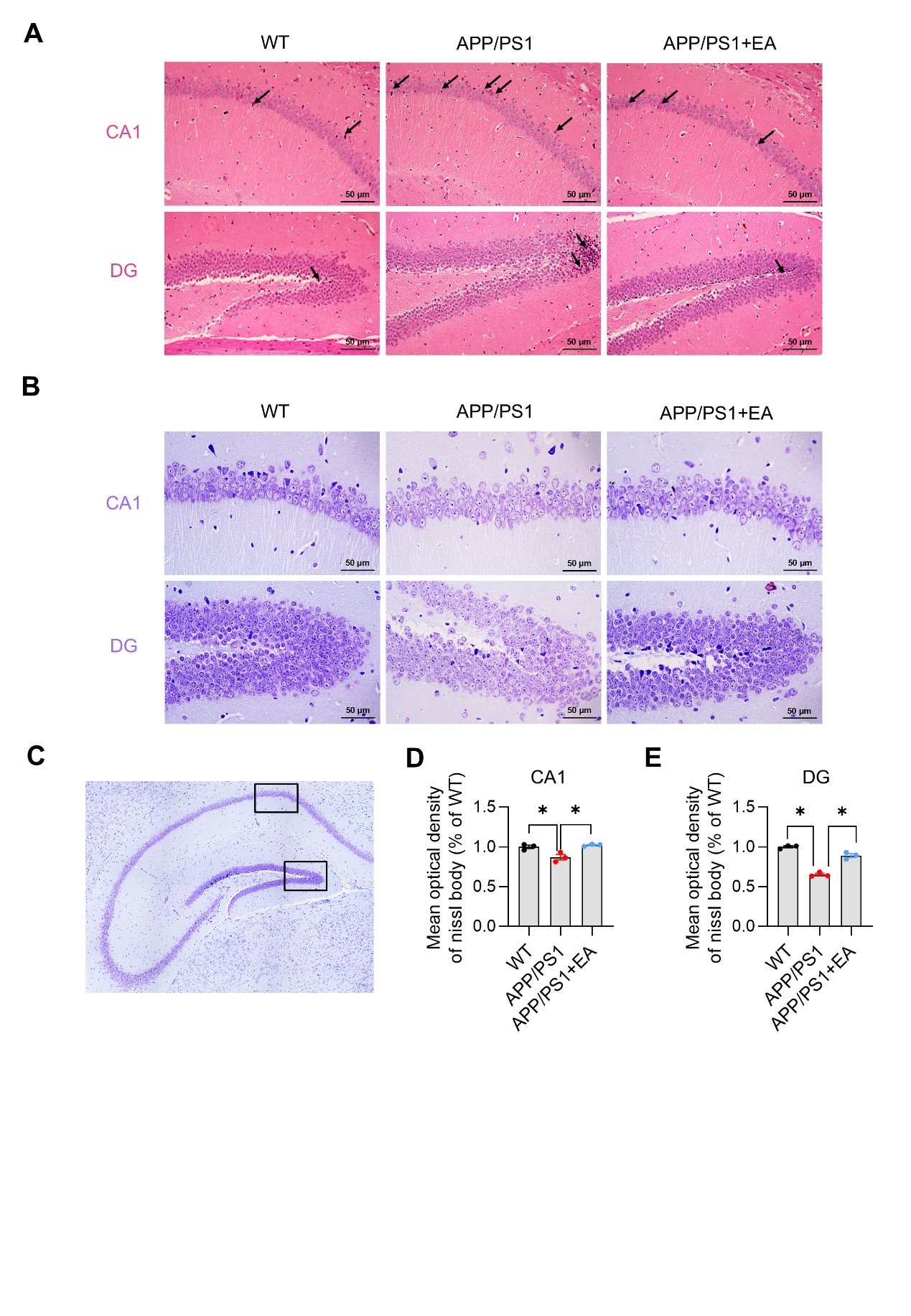


**sFig.1 Electroacupuncture prevents hippocampal neuronal degeneration in APP/PS1 mice.** (A) Representative images of hippocampal CA1 and DG regions measured by HE staining (200 x). Scale bar: 50 μm. (B) Representative images of hippocampal CA1 and DG regions measured by Nissl staining (200 x). Scale bar: 50 μm. (C) Illustration of a mouse brain section. Nissl staining was observed in the region depicted by the rectangular box. (D-E) The mean optical density of Nissl bodies of hippocampal CA1 and DG regions. Data are expressed as mean ± SEM (n = 3 per group). **p* < 0.05 between groups. (one-way ANOVA followed by *Tukey* test).


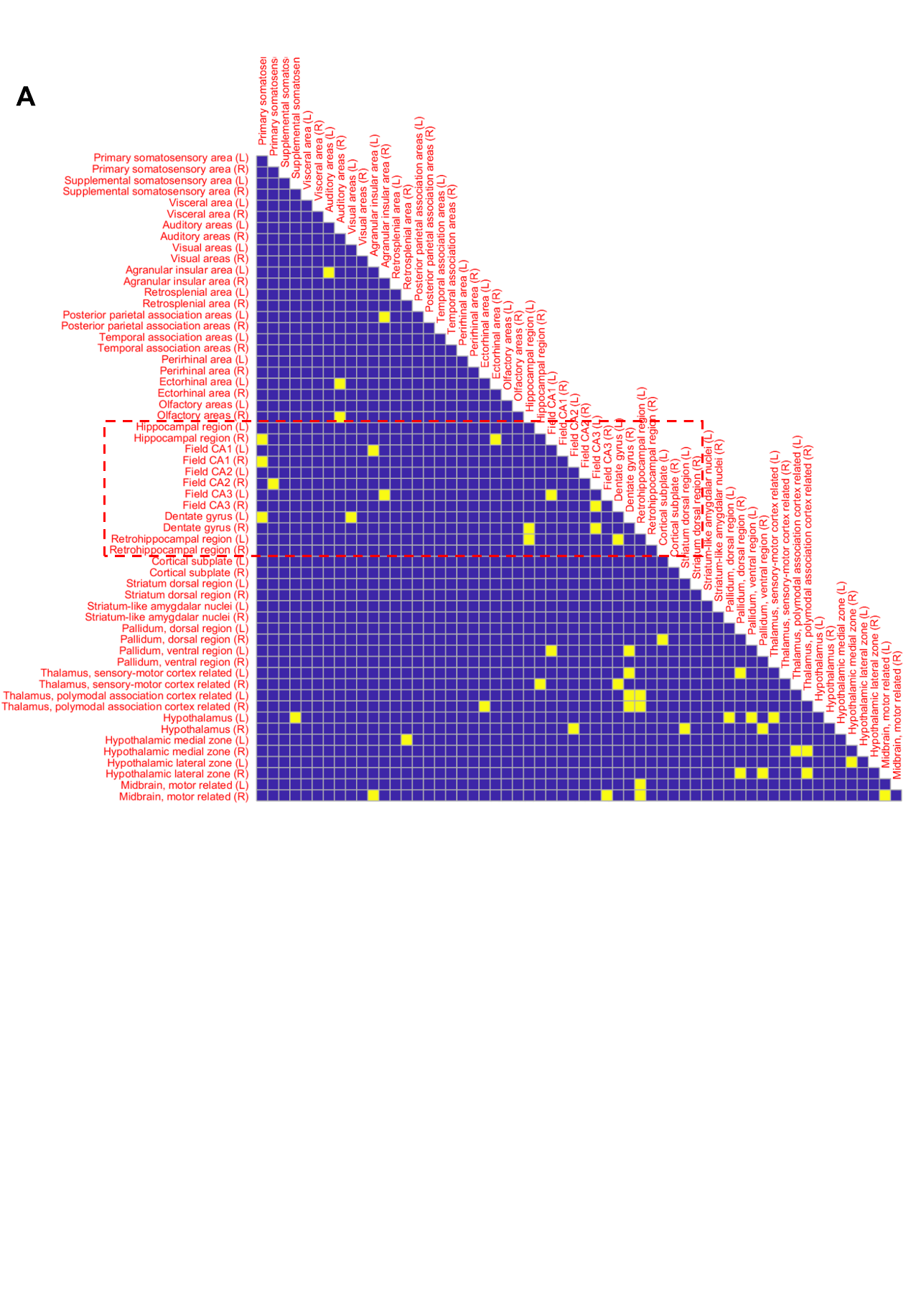


**sFig.2 Binary matrix of significant functional connectivity derived from MATLAB analysis.**
